# Composted invasive plant *Ageratina adenophora* enhanced barley (*Hordeum vulgare*) growth and soil conditions

**DOI:** 10.1371/journal.pone.0275302

**Published:** 2022-09-29

**Authors:** Hai Liu, Qing Zhao, Yanhua Cheng

**Affiliations:** 1 College of Business Administration, Guizhou University of Finance and Economics, Guiyang, Guizhou, China; 2 College of Resources and Environment, Southwest University, Chongqing, China; 3 College of Agriculture, Yangtze University, Jingzhou, Hubei, China; Hunan Agricultural University, CHINA

## Abstract

*Ageratina adenophora* originating from central America has flooded forests, pastures, and farmland in more than 40 tropical and subtropical countries, causing huge ecological disasters and economic losses. In this paper, we intended to use a complex inoculum composed of *Pseudomonas putita* and *Clostridium thermocellum* to *in-situ* compost *A*. *adenophora* debris and then to compare the phytotoxicity of extracts from uncomposted and composted *A*. *adenophora* (UCA and CA respectively) to barley seed germination and young seedling growth. A field experiment was finally conducted to reveal the effects of UCA and CA on barley nutrients uptake, yield, grain quality, soil enzyme activities, microbial biomass and biodiversity. *In-situ* composting sharply decreased 4,7-dimethyl-1-(propan-2-ylidene)-1,4,4a,8a-tetrahydronaphthalene- 2,6(1H,7H)-dione(DTD) and 6-hydroxy-5-isopropyl-3,8-dimethyl-4a,5,6,7,8,8a-hexahydronaphthal en-2(1 H)-one(HHO) from 2096.3 and 743.7 mg kg^-1^ in uncomposted *A*. *adenophora* to 194.4 and 68.19 mg kg^-1^ in composted *A*. *adenophora*. UCAE showed negative influences on seed germination performances (except lower rates on germination percentage). The mechanism may be the inhibition of bio-macromolecules hydrolysis (including proteins, starch, and phytin) in endosperms and their hydrolysates for forming new plants. CAE promoted seed germination and seedling growth, increased chlorophyll levels in leaves, and stimulated dehydrogenase and nitrate reductase activities in plants, while UCAE got opposite performance. Compared with chemical fertilizers, application of CA in combination with chemical fertilizers significantly improved plant nutrient uptake (nitrogen, phosphorus, and potassium), yield, grain quality, quantity of 16S rDNA sequences, richness and diversity of bacterial communities in contrast to UCA which behaved otherwise. Taken together, the use of the microbial agent to *in-situ* compost *A*. *adenophora* may be an effective approach for agricultural use of *A*. *adenophora* debris as a plant-friendly organic fertilizer, being undoubtedly worth advocating.

## Introduction

*Ageratina adenophora* is a member of the genus *Eupatorium* belonging to the daisy family Compositae. As a perennial malignant weed native to Costa Rica in Central America, *A*. *adenophora* is difficult to remove due to its strong vitality and sexual and asexual reproduction [1]. Since spread into China across the China-Myanmar border in the 1940s, this invasive plant has now become everywhere in southwestern China, causing serious ecological disasters and tremendous economic losses to local agriculture, animal husbandry, and forestry. Approximately 500 billion dollars of annual economic loss was confirmed from destroying the ecological function of forests, arable lands, and meadows [2]. *A*. *adenophora* is convinced as the most problematic harmful invasive plant in China.

Most invasive plants release allelochemicals to the surrounding environments through root exudation, rainfall leaching, and residue composting, which affects the survival of neighboring plants and at the same time places themselves in a competitive advantage in expanding their living space and population [3]. For example, more than 100 chemical substances have been identified from *A*. *adenophora* plants as well as their rhizospheres. The majority of these substances are monoterpenes, oxime, triterpenoids, phenylpropanoids, flavonoids, and their derivatives. Of those, 4,7-dimethyl-1-(propan-2-ylidene)-1,4,4a,8a-tetra-hydronaphthalene-2,6(1H,7H)-dione (DTD) and 6-hydroxy-5-isopro pyl-3,8-dimethyl-4a,5,6,7,8, 8a-hexahydronaphtha len-2(1 H)-one (HHO) account for a large proportion, which are toxic to animals, microbes, and plants. They are probably the main phytotoxic allelochemicals released by *A*. *adenophora* to the environment [4].

Previous studies indicate that *A*. *adenophora* extracts have strong inhibitory effects on seed germination and plant growth, such as pepper, eggplant, pea, tomato, wheat, rice, and ryegrass [5, 6]. *A*. *adenophora* litters release a large amount of allelochemicals into the environment and inhibit the growth of surrounding plants, leading to the rapid formation of its single dominant population [7]. Moreover, as *A*. *adenophora* has strong vitality, both vegetative and reproductive organs, the eradication of *A*. *adenophora* confronts unimaginable obstacles. Resourceful utilization of *A*. *adenophora* plant residues is a practical way of controlling its diffusion. For a long time, multiple products were attempted to make from *A*. *adenophora* plants, such as biofuels [8], biochar [9], chlorogenic acid [10], and phytogenic pesticides [11]. Nonetheless, few were proved practical and economical. Additionally, the attempts of utilizing *A*. *adenophora* also failed in paper-making industries [12], animal feed [13], and fuelwood preparation [14], which lies in the fact that *A*. *adenophora* has short fiber in length, and contains more than 100 kinds of toxins and low calorific value. That is why the new ways of utilizing *A*. *adenophora* are still under desideration.

In crop cultivation, both organic fertilizer application and straw returning to fields functioned in improving soil physical and chemical properties, as well as enhancing soil nutrients supply and crop yield and quality [15, 16]. Fan and Huang [17] reported that *A*. *adenophora* grows rapidly, has large biomass, and is also rich in nitrogen (N), phosphorus (P), potassium (K), and trace elements. Thus, *A*. *adenophora* would be a good source of organic fertilizers if the toxic allelochemicals that inhibit plant growth are removed and the reproductive organs are inactivated. In the natural composting process, the toxic allelochemicals in *A*. *adenophora* inhibit microbial activity, resulting in prolonged and poor composting efficiency. Meanwhile, the seeds and vegetative reproductive organs cannot be completely inactivated. Previous studies have revealed that polycyclic aromatic hydrocarbons (including benzene, naphthalene, phenanthrene, and anthracene) can be degraded by *Pseudomonas*, *Alcaligenes denitrificans*, *Arthrobacter polychromogenes*, *Cycloclasticus*, *Acinetobacter calcoaceticus*, and *Nocardia*; vanillin and coumarin by *Penicillum chrysogenum*; and tannin by *Aspergillus ustus* and *Aspergillus niger*. Consequently, these microorganisms can be used to degrade the toxic allelochemicals in *A*. *adenophora* debris for the harmless treatment and resource utilization of this plant [18].

*A*. *adenophora* covers more than 15% of the land area in Liangshan prefecture, Sichuan province, China, leading to serious agricultural, forestry, and ecological problems. In the artificial removal of this invasive plant, a large amount of plant residues need to be properly handled. In this paper, we employed the grain crop barley, which was widely grown in the local area every year, as the test crop to explore the performances of seed germination and seedling growth in response to *A*. *adenophora* extracts, and to evaluate the effects of composted and uncomposted *A*. *adenophora* (CA and UCA, respectively) on barley yield and quality in the field. The results obtained may provide some scientific information for effectively controlling and at the same time utilizing the plant debris as an organic fertilizer resource.

## Materials and methods

### On-site composting of *A*. *adenophora*

Based on actual artificial weeding practice, we collected the aerial parts of *A*. *adenophora* plants in five randomly selected sites and thereafter conducted the *in situ* composting in March 2019 and 2020 in Mianning County, Liangshan prefecture of China (28°30′37″N, 102°07′55″E). The collected *A*. *adenophora* stems and shoots were cut into segments 2–3 cm in length, then piled in a width of 2–3 m and height of 1–1.5 m with a plastic film covered. Each layer (approximately 50 cm per layer) of the piled materials (leaves, stems, and shoots) was evenly sprinkled with the mixed bacterial inoculant composed of *Clostridium thermocellum* and *Pseudomonas putita* (10^9^ CFU g^-1^), lime, and urea. The weight ratio of materials, bacterial inoculant, lime, and urea was 1000: 2: 1: 1.5 [19]. The mixed bacterial inoculant was self-prepared. Adding lime and urea was to neutralize the organic acids produced during the composting process, decrease the ratio of carbon to nitrogen [20], and finally well compost the toxic substances in the plant debris. In addition, the covered plastic film was to hold moisture and heat produced when the plants were composted. About 40–50 days later *A*. *adenophora* was fully composted and ready for use.

### *A*. *adenophora* extracts

Both UCA and CA samples were individually collected from the above-mentioned five randomly selected sites, oven-dried, weighed, and ground to pass through a 1-mm sieve. Five UCA and five CA samples were prepared. The chemical properties of UCA and CA were determined, including pH, organic matter, nitrogen, phosphorus, potassium, humic, DTD and HHO. pH (1:2.5) was detected with a pH metre (FE20, Mettler Toledo Co., Ltd., Shanghai, China). K_2_Cr_2_O_7_ oxidation method was employed in organic matter determination. Humic acid was measured according to national standard GB/T 11957–2001. Then we digested UAC and CA samples with H_2_SO_4_-H_2_O_2_. N, P, and K in the digests were determined with the methods of respectively Kjeldahl distillation, molybdenum blue colorimetry and flame photospectrometry [21]. The concentrations of DTD and HHO in aqueous extractions of the five UCA and five CA samples were detected with a L-2000 high-performance liquid chromatography (Eclipse Plus, USA) by referring to [22] in detail. The analysis column GS-310 (C18 column, inner diameter × length = 4.6 mm × 150 mm) used in conjunction was purchased from Japan Analytical Industry Company, Tokyo, Japan. The mobile solvent system was composed of methanol and water (70:30, volume) at 1 mL min^−1^ and 1.5 MPa. 10 mL aliquots were injected into HPLC system, the UV detector wavelength and column temperature were respectively 254 nm and 30 ℃, and retention time for DTD and HHO detection was respectively 3.1 min and 8.2 min. The standards of DTD and HHO were obtained from Yangzhou University in China. The recovery rates for DTD and HHO were respectively 92.7% and 94.5%.

Afterward, 10.0 g of each were soaked in 1 L distilled water, intermittently stirred at 37 ℃ for 12 h, and then filtered to formulate 1% mother solutions. The chemical analysis demonstrated that each liter of UCA extract (UCAE) contained DTD 2.27 g, HHO 0.74 g, N 0.22 g, P 0.14 g, K 0.49 g on average, while each liter of CA extract (CAE) contained DTD 0.19 g, HHO 0.06 g, N 1.63 g, P 0.33 g, K 0.81 g on average. In order to avoid the extra effect of higher nutrient contents in CA extract on seedling growth, we added appropriate amounts of (NH_4_)_2_SO_4_, NH_4_H_2_PO_4_, and K_2_SO_4_ to UCAE, thus to equal the nutrient concentrations in both UCAE and CAE. After diluted with deionized water, Solutions with 0 (control), 1.00, 10.0, and 100 mg UCAE (or CAE) L^-1^ were serially prepared for applying in the follow-up experiments.

### Experimental design and measurement items

#### Seed germination and seedling culture tests

The seed germination test was conducted in Plant Nutrition Laboratory of College of Resources and Environment, Southwest University in June, 2020. Uniform and healthy barley seeds (cultivar Lanmai 10) were disinfected with 1.0% H_2_O_2_ for 1 min, then washed with deionized water, and placed in deionized water and solutions at various concentrations of UCAE and CAE (1, 10, and 100 mg L^-1^) for 24 h at 25°C. Afterward, 50 fully-imbibed seeds were selected and placed in a 15-cm-diameter petri dish with three layers of filter paper at the bottom. These seeds were cultured at 25°C with 10000 lx of light density in a light-dark cycle of 12 h/12 h. During the seed germination and seedling culture periods, the corresponding solutions (deionized water, various concentrations of UCAE and CAE) were daily added as needed. There were five replicate germination Petri dishes for each concentration treatment.

Taking radicle length ≥ 1 mm as the standard, the number of germinated seeds was recorded every 24 hours. The germination was considered completed when no germination occurred within a day. Then the germination percentage, as well as germination and vigor indexes, were calculated according to the following formulas [23]:

$$\text{Seed}\;\text{germination}\;\text{percentage}=\left(\text{number}\;\text{of}\;\text{germinated}\;\text{seeds}/\text{total}\;\text{number}\;\text{of}\;\text{tested}\;\text{seeds}\right)\times 100\%$$ (1)

Seedgerminationindex=∑Gt/Dt(Gt,germinationdays;Gt,numberofseedsgerminatedperdayDt) (2)

Seedvigorindex=GI×S(GI,seedgerminationindex;S,seedlingheightincm) (3)

After soaked in UCAE and CAE solutions for 48 h, some radicle-elongated seeds were randomly selected and digested with H_2_SO_4_-H_2_O_2_, and extracted with deionized water, respectively. P in the digests (insoluble P in seeds) and the water (soluble P in seeds) were analyzed by the molybdenum blue colorimetry [24]. Meanwhile, the colorimetric anthrone-perchloric acid method and anthrone colorimetry were employed to determine starch and soluble sugar levels in the seeds [25]. Proteins and free amino acids in the seeds were analyzed by Coomassie brilliant blue method and ninhydrin colorimetry [26].

Simultaneously we conducted the seedling culture test in the laboratory as well, we sowed 15 germinating barley seeds in plastic pots (diameter × height = 15 cm × 15 cm) filled with sterile quartz sands (ϕ = 1–1.5 mm), and watered with Hoagland nutrient solution containing various concentrations of UCAE and CAE (0, 1, 10, 100 mg·L^-1^) every other day, respectively. The seedlings were cultured for 21 days at 25±1 ℃ with 10000 lx light intensity in a 12 h/12 h light-dark period. There were five replication pots for each concentration treatment. The biomass, height, and maximum root length of barley seedlings were recorded after growing the seedlings for three weeks. Meanwhile, dehydrogenase activity (DHA) in the roots was analyzed by 2,3,5-triphenyltetrazolium chloride (TTC) method [27] and nitrate reductase activity (NRA) in the first fully-expanded leaves by sulfonamide-naphthylamine colorimetry [28]. The first fully-expanded leaves were extracted with acetone and analyzed for chlorophyll spectrophotometrically at 652 nm [29]. In DHA measurement, we sampled 1 g fresh root tips (1 cm length) and immersed in phosphate buffer solution (pH 7.0, 20 mL) with 0.2% TTC added, and then placed in totally dark at 30 ◦C for 3 h, then we used ethyl acetate to extract triphenyl tetrazolium formazan formed in the incubation process and colormetric at 485 nm [27]. To detect NRA in leaf, 2 g leaves were ground and dispersed in 10 mL phosphate buffer solution (pH = 7.5) with adding nicotinamide adenine dinucleotide (2.0 mg mL^-1^), then the above mentioned solution was centrifuged for 20 min at the speed of 6000 rpm, the supernatants was cultured at 25 ◦C for 1 h after adding 0.1 mol L^−1^ KNO_3_, α-neamine was employed to react with NO_2_^-^ generated during the culture process to produce a red azo compound, then we conducted the measurement spectrophotometrically at 540 nm.

#### Field experiment

The two-year field experiment was conducted during Nov.1, 2019 to Apr. 10, 2020, and Nov. 5, 2020 to Apr. 13, 2021, in Henglu Village (28°30’30"N, 102°7’49"E) of Mianning County, Liangshan Prefecture, China (1822 m above sea level). Barley and flue-cured tobacco crop rotation system was implemented in Henglu Village. The barley seeds were directly sowed in early November. Field management, including wedding, irrigation, pest control, etc. was the same as the local barley cultivation. The local climate is dominated by subtropical monsoon with an annual average temperature of 17.1°C, annual sunshine hour of 2431 h, and annual precipitation of 1087.5 mm. Basically the weather conditions did not vary too much in both years, and had little impact on data accuracy of our field experiment. The experimental soil (Eutric Regosol, UAO Soil Taxonomic System) was derived from alluvial deposits in the Triassic period, had a loam texture with pH 5.53, and amounted at 22.75 g kg^-1^ of organic matter (OM), 1.73 g kg^-1^ of total N (TN), 0.66 g kg^-1^ of total P (TP), 19.63 g kg^-1^ of total K (TK), 186.11 mg kg^-1^ of 1N NaOH-hydrolyzed N (AN), 46.92 mg kg^-1^ of Olsen P (AP), and 169.18 mg kg^-1^ of 1N CH_3_COONH_4_-exchangeable K (AK).

The experimental treatments included: no fertilizer (CK), chemical fertilizer (CF), CF+UCA, and CF+CA. UCA was prepared from fresh *A*. *adenophora* plants by drying under the sun. In the CF, CF+UCA, and CF+CA treatments, the same amounts of nutrients were applied (120 kg N ha^-1^, 80 kg P_2_O_5_ ha^-1^, and 60 kg ha^-1^, supplied as urea, calcium superphosphate, potassium chloride, UCA, and CA, respectively). Half of N was provided by UCA or CA and the residual N, P, and K were supplemented by chemical fertilizers in the UCA and CA treatments. All UCA, CA, P, and K fertilizers were fertilized as base fertilizer. N in the base fertilizers accounted for 70% of the total N applied and another N was side-dressed in the tillering stage. A completely randomized block design was used to lay out each treatment with four replications. The field plot area was 31.35 m^2^ (4.75 m×6.6 m) and approximately six million barley seeds were sown in each hectare.

In early April of 2020 and 2021, before harvesting we used five-piont sampling method to get straw and grain samples in each plot separately. Then the straw samples and grain samples were individually digested with H_2_SO_4_-H_2_O_2_ for the analysis of N by the Kjeldahl method [30], P by the molybdenum blue colorimetry [31], and K by the flame photometry [32]. Starch and protein contents in grain were determined by the colorimetric anthrone-perchloric acid method [25] and Coomassie brilliant blue method [33], respectively. After sampling we harvested the aboveground part of barley plants, and employed a smart thresher to finish barley grain threshing. It is worth noting that both of the harvesting and threshing procedures were conducted per plot separately, and the biomass and grain yield of each plot were separately weighed and recorded.

In early April 2021, the rhizosphere soil of barley plant was sampled from each plot of all treatments. Part of freshly collected soil samples were used to measure dehydrogenase activity, as well as the soil microbial biomass C and N (SMBC and SMBN) and to detect the bacterial rDNA sequences, the measured indexes included quantity of 16S rDNA sequences, richness indexes of Ace and Chao, as well as Shannon’s diversity index. The other part soil samples were air-dried for analyzing the activities of urease and invertase. To measure the soil dehydrogenase activity, the soil samples were incubated with 1% TTC solution, and triphenyl formazone (TF) generated was colorimetrically detected at 485 nm [34]. In measuring the soil urease activity, we employed a modified indophenol reaction [35], extracting ammonium with the mixed 1 N KCl and 0.01 N HCl solution, and colorimetric NH_4_^+^. To detect the soil invertase activity, the soil samples were raised in sucrose solution for 24 h at 37℃, and measured spectrophotometrically at 508 nm [35]. To detect SMBC and SMBN, the soil samples were fumigated with chloroform and extracted with K_2_SO_4_, thus to analyze the SMBC by K_2_Cr_2_O_7_ oxidation and SMBN by indophenol blue spectrophotometry [36]. The quantity of 16S rDNA sequences, as well as the richness, and diversity indicators, were processed in the sequence of amplification and pooling, which were conducted in Majorbio Biotech Co., Ltd (Shanghai, China).

### Statistical analysis

All data obtained in each experiment were subjected to one-way ANOVA analysis using SPSS 24.0 (IBM Corporation, NC, USA). The least significant difference (LSD) was used to compare the treatment effect at the significant level of *P*<0.05.

## Results and analysis

### Chemical properties of uncomposted and composted *A*. *adenophora*

As is shown in Table 1, pH, nitrogen and potassium content of composted *A*. *adenophora* were higher than that of uncomposted *A*. *adenophora* by 16.5%, 22.5% and 70.3% respectively. The contents of organic matter and phosphorus did not vary too much before and after composted. Most importantly, contents of DTD and HHO decreased from 2096.3 and 743.7 mg kg^-1^ to 194.4 and 68.19 mg kg^-1^ respectively through composting, approximately ten times lower in composted *A*. *adenophora* than that in uncomposted *A*. *adenophora*.

**Table 1:** Chemical properties of uncomposted and composted *A*. *adenophora*.

Indexes	Uncomposted *A*. *adenophora*	Composted *A*. *adenophora*
pH	6.11±0.02 b	7.12±0.01 a
Organic matter/%	93.64±5.08 a	90.11±3.79 a
Nitrogen/%	2.18±0.14 b	2.67±0.16 a
Phosphorus/%	0.60±0.13 a	0.78±0.12 a
Potassium/%	1.55±0.06 b	2.64±0.09 a
Humic acid/%	nd	8.12±0.88
DTD*/(mg kg^-1^)	2096.3±315.4 a	194.4±70.53 b
HHO*/(mg kg^-1^)	743.7±80.92 a	68.19±9.74 b

*DTD and HHO represented 4,7-dimethyl-1-(propan-2-ylidene)-1,4,4a,8a-tetrahydronaphthalene-2,6(1H,7H)- dione and 6-hydroxy-5-isopropyl-3,8-dimethyl-4a,5,6,7,8,8a-hexahydronaphthalen-2(1 H)-one respectively; nd means not detected; Different letters in the same column indicate significant difference at *P*<0.05.

### Seed germination properties as affected by *A*. *adenophora* extracts

**Fig 1:**
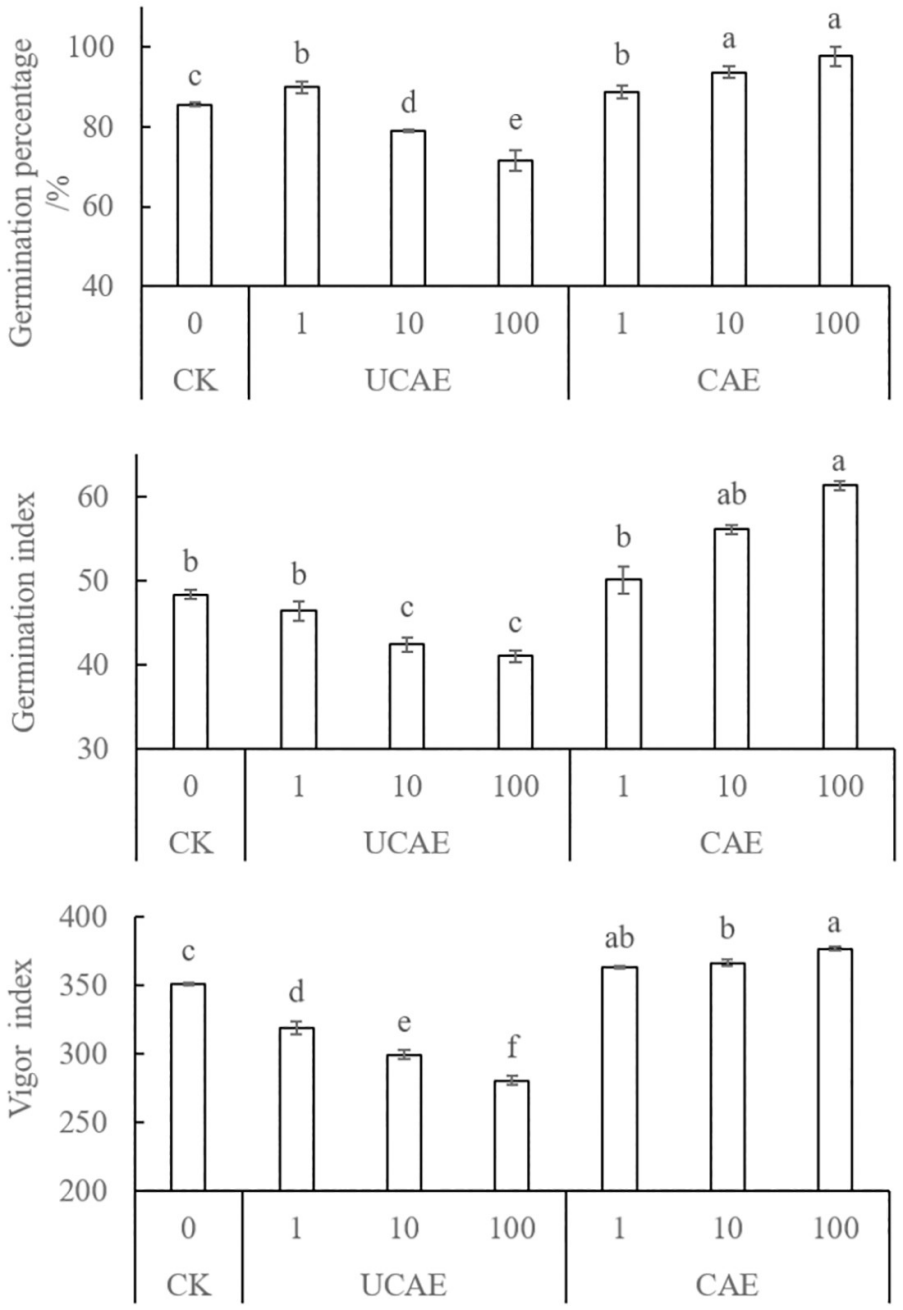
Germination properties of barley seeds treated with uncomposed and composted *A*. *adenophora* extracts (UCAE and CAE, respectively). On each column, data marked with different small letters are significantly different at *P* < 0.05. UCAE = uncomposted *A*. *adenophora* extract; CAE = composted *A*. *adenophora* extract. Unit for UCAE and CAE is mg L^-1^.

As shown in Fig 1, low concentration UCAE (1 mg L^-1^) promoted barley germination percentage, but decreased germination index and vigor index in comparison with the blank control (CK). With the concentration upgoing, an obvious decreasing trend could be observed in the change of germination percentage, germination index, and vigor index. On the other hand, these three parameters increased with CAE concentrations. For example, germination percentage, germination index, and vigor index were increased from 85.66%, 48.35, and 351.21 in CK to 97.73%, 61.31, and 376.95 in the 100 mg CAE L^-1^ treatment, respectively.

### Imbibed seed inclusions as affected by *A*. *adenophora* extracts

Compared with CK, the imbibed seeds that received CAE contained higher free amino acids, soluble sugars, and soluble P (the differences in free amino acids and soluble P were not significant between CK and 1.00 mg CAE L^-1^, Table 2). There were lower proteins, starches, and insoluble P in the imbibed seeds (not significant in proteins among CK, 1.00, and 10.0 mg CAE L^-1^ and in insoluble P between CK and 1.00 mg CAE L^-1^). In contrast, UCAE (particularly at high concentrations) increased proteins, starches, and insoluble P but decreased free amino acids, soluble sugars, and soluble P in the imbibed seeds.

**Table 2:** Effects of the extracts from *A*. *adenophora* on inclusions in germinating seeds (mean ± SD; %).

Treatments	Extract concentrations mg L^-1^	Free amino acids	Protein	Soluble sugar	Starch	Soluble P	Insoluble P
CK	0.00	2.10±0.21 c	9.79±0.26 bc	12.69±1.13 b	60.28±5.19 b	16.37±2.02 b	0.54±0.11 ab
UCAE	1.00	2.05±0.23 c	10.85±0.05 ab	11.83±1.61 bc	60.87±4.71 b	14.58±1.64 c	0.60±0.09 ab
	10.0	1.87±0.17 cd	12.06±0.21 a	10.59±0.76 bc	61.46±6.09 b	12.39±1.78 c	0.62±0.19 ab
	100	1.26±0.29 d	12.94±0.39 a	8.57±1.39 c	75.89±5.25 a	10.12±1.51 c	0.72±0.07 a
CAE	1.00	2.13±0.11 c	9.58±0.03 bc	17.77±0.44 a	41.48±4.14 c	16.71±1.29 b	0.49±0.06 bc
	10.0	3.28±0.23 b	9.16±0.15 bc	18.19±0.81 a	41.26±5.89 c	18.39±1.34 a	0.43±0.12 c
	100	4.69±0.17 a	8.27±0.05 c	19.23±1.62 a	40.73±5.41 c	19.27±1.28 a	0.36±0.07 c

CK = blank control, UCAE = UCA (uncomposted *A*. *adenophora*) extracts, CAE = CA (composted *A*. *adenophora*) extracts, different letters in the same column indicate significant difference at *P*<0.05.

### Growth and selected physiological indexes of young seedlings

Compared with CK, UCAE significantly inhibited the growth of barley seedlings, with biomass decreased by 0.41–28.80%, seedling height by 6.57–23.01%, and root length by 29.89–50.82% (Table 3). On the other hand, the addition of CAE to culture substrates increased these growth parameters although not significant at 1.00 mg CAE L^-1^.

**Table 3:** Effects of extracts from *A*. *adenophora* on the growth and selected physiological indexes of barley seedlings (mean ± SD).

Treatments	Concentration mg L^-1^	Biomass g plate^-1^	Seedling height cm	Maximum root length cm	DHA* in roots mg TTF g^-1^ h^-1^	NRA* μg·g^-1^ h^-1^	Chlorophyll μg g^-1^
CK	0	7.36±0.03 c	9.43±0.72 c	3.68±0.41 b	1.25±0.02 c	4.21±0.06 bc	1.91±0.02 c
UCAE	1.00	7.33±0.07 c	8.81±0.44 cd	2.58±0.12 c	1.28±0.03 c	4.32±0.03 bc	1.78±0.04 d
	10.0	5.33±0.11 d	8.18±0.39 d	2.32±0.39 c	0.81±0.05 d	3.66±0.05 c	1.59±0.05 e
	100	5.24±0.01 d	7.26±0.23 e	1.81±0.23 d	0.36±0.08 e	3.43±0.06 c	1.28±0.02 f
CAE	1.00	7.48±0.04 c	9.94±1.07 c	2.77±0.13 c	1.38±0.04 bc	4.72±0.03 b	1.91±0.08 c
	10.0	9.39±0.06 b	11.36±1.16 b	3.38±0.59 b	1.46±0.02 b	4.88±0.07 b	2.48±0.07 b
	100	9.96±0.19 a	12.87±1.61 a	4.24±0.23 a	1.67±0.03 a	5.39±0.18 a	2.79±0.09 a

CK = blank control, UCAE = UCA (uncomposted *A*. *adenophora*) extracts, CAE = CA (composted *A*. *adenophora*) extracts, DHA = dehydrogenase activity, and NRA = nitrate reductase activity. Different letters in the same column indicate significant difference at *P*<0.05.

Dehydrogenase activity in the roots and chlorophyll in the leaves showed a downward trend, while nitrate reductase activity in the leaves was maintained unchanged with UCAE concentrations increased (also Table 3). These three physiological indexes exhibited a positive dose-dependent increase after CAE was added to culture substances.

### Nutrients uptake and grain yield and quality

As shown in Table 4, the indicators of nutrients uptake, grain yield and quality had similar change trends among treatments in the years of 2020 and 2021, and no yearly difference occurred. Compared with no fertilizers, CF significantly increased barley nutrients uptake by respectively 25.21% (N), 16.07% (P), 15.61% (K) in 2020, and 26.03% (N), 29.61% (P), 15.86% (K) in 2021. As a result, the grain yield was increased by 60.65% and 66.09% respectively in 2020 and 2021.

**Table 4:** Effects of uncomposted and composted *A*. *adenophora* on barley nutrient uptake and grain yield and quality.

Year	Treatments	Nutrient uptake/ (kg ha^-1^)			Grain yield/ (kg ha^-1^)	Grain quality/ %	
		N	P	K		Proteins	Starches
2020	CK	203.88±18.90 c	45.44±3.66 c	226.26±10.72 c	2881.43±93.60 c	9.21±0.12 c	60.39±1.66 b
	CF	259.26±11.99 b	52.74±3.25 b	261.57±9.52 b	4628.93±106.95 b	10.46±0.13 b	69.78±2.42 a
	CF+UCA	255.27±9.57 b	51.71±2.16 b	248.08±13.03 bc	2850.94±88.77 c	8.24±0.08 d	61.41±1.39 b
	CF+CA	296.64±18.31 a	60.24±4.50 a	299.75±12.81 a	5568.74±164.83 a	11.69±0.10 a	67.74±1.53 a
2021	CK	207.71±14.22 c	41.71±3.23 c	211.57±10.95 c	2859.81±88.92 c	9.36±0.09 c	61.17±2.15 b
	CF	261.78±12.33 b	54.06±5.13 b	245.13±9.64 b	4749.88±108.94 b	10.55±0.09 b	68.31±1.84 a
	CF+UCA	256.41±12.90 b	53.61±5.12 b	242.84±10.78 b	2793.31±81.94 c	7.64±0.08 d	60.59±2.09 b
	CF+CA	308.77±19.37 a	63.60±5.41 a	306.30±12.88 a	5674.93±152.19 a	11.61±0.08 a	67.21±1.33 a

CK = blank control, CF = chemical fertilizer, CF+UCA = chemical fertilizer plus uncomposted *A*. *adenophora*, CF+CA = chemical fertilizer plus composted *A*. *adenophora*. Different letters in the same column indicate significant difference at *P*<0.05.

Compared with CF, CF+UCA did not vary significantly in nutrients uptake, while decreased grain yield by 38.41% and 41.19% respectively in 2020 and 2021 (also Table 4). By contrast, CF+CA increased plant nutrients uptake by 14.42% and 17.95 for N, 14.22% and 17.65% for P, 14.60% and 24.95% for K in 2020 and 2021. With the increase in nutrient absorption, the grain yield was increased by 20.30% and 19.48% in 2020 and 2021 respectively in the CF+CA treatment.

The protein content in barley grains changed in the sequence: CF+CA > CF > CK > CF+UCA (also Table 4). The grains contained similar starches in the CK and CF+UCA treatments (60.39–61.41% in 2020, 60.59–61.17% in 2021), much lower than those in CF and CF+CA (67.74–69.78% in 2020, 67.21–68.31% in 2021), and the difference was not significant between CF and CF+CA.

### Soil enzyme activity and microbial biomass

The application of uncomposted *A*. *adenophora* (CF+UCA) significantly decreased soil enzyme activities of urease, invertase, and dehydrogenase (Table 5), as well as SMBC and SMBN compared with CK, CF, and CF+CA. Conversely, applying CA significantly increased all the indicators in comparison with CK and CF.

**Table 5:** Effects of uncomposted and composted *A*. *adenophora* on soil enzyme activities.

Treatments	Urease/μg NH_4_^+^-N g^-1^ h^-1^	Invertase/μg Glu g^-1^ h^-1^	Dehydrogenase/μg TTF g^-1^ h^-1^	SMBC/mg kg^-1^	SMBN/mg kg^-1^
CK	13.23±2.41 c	13.77±3.69 b	5.19±1.54 b	65.71±3.43 c	16.62±1.14 c
CF	20.39±1.47 b	16.44±2.78 b	4.87±1.67 b	80.89±5.43 b	20.11±2.22 b
CF+UCA	7.49±1.58 d	7.48±1.69 c	2.79±1.44 c	40.31±2.68 d	11.19±1.68 d
CF+CA	25.37±2.13 a	23.97±3.31 a	7.71±1.48 a	120.8±7.76 a	25.31±3.44 a

CK = blank control, CF = chemical fertilizer, CF+UCA = chemical fertilizer plus uncomposted *A*. *adenophora*, CF+CA = chemical fertilizer plus composted *A*. *adenophora*. SMBC = soil microbial biomass carbon, SMBN = soil microbial biomass nitrogen. Different letters in the same column indicate significant difference at *P*<0.05.

### Quantity of 16S rDNA sequences, richness and diversity of bacterial communities

Averagely 13972, 15656, 11063, and 15948 16S rDNA sequences were picked up through MiSeq illumina sequencing in soils from CK, CF, CF+UCA, and CF+CA. CF+CA owned the greatest quantity of 16S rDNA sequences, significantly higher than CK and CF+UCA, while not significantly different with CF. Nevertheless, applying CF+UCA greatly decreased the number of 16S rDNA sequences, significantly lower than other treatments (Table 6). The soil bacterial communities owned the highest Ace richness indexes, Chao, and Shannon’s diversity index in CF+CA, while the lowest ones in CF+UCA. CF and CF+CA did not vary significantly, and both of them were significantly higher than CK and CF+UCA.

**Table 6:** Quantity of 16S rDNA sequences, richness and diversity of bacterial communities in different treatments.

Treatments	Quantity of 16S rDNA sequences	Richness indexes		Shannon’s diversity index
		Ace	Chao	
CK	13972±1247 b	943.2±60.19 b	936.3±55.43 b	3.25±0.17 b
CF	15656±1149 a	1044±97.36 a	1028±85.11 a	4.79±0.36 a
CF+UCA	11063±1077 c	732.4±55.61 c	717.4±48.15 c	2.11±0.17 c
CF+CA	15948±1328 a	1138±114.7 a	1149±101.3 a	5.18±0.32 a

CK = blank control, CF = chemical fertilizer, CF+UCA = chemical fertilizer plus uncomposted *A*. *adenophora*, CF+CA = chemical fertilizer plus composted *A*. *adenophora*. Different letters in the same column indicate significant difference at *P*<0.05.

## Discussion

Mechanical and manual removal of *A*. *adenophora* generated piled plant debris, which needs to be treated innocuously and utilized as natural resources. The prerequisite of using the debris as raw materials of producing organic fertilizer is to completely degrade allelochemicals toxic to plants and inactivate all the reproductive organs, including roots, stems, shoots, and seeds. DTD and HHO are the key allelochemicals released from *A*. *adenophora* during the whole growth period. In our study, DTD and HHO concentration sharply decreased from 2096.3 and 743.7 mg kg^-1^ in uncomposted *A*. *adenophora* to 194.4 and 68.19 mg kg^-1^ in composted *A*. *adenophora* separately, this result is consistent with reference [22]. The decomposition of DTD and HHO realized the first-step forward in resourceful utilization of *A*. *adenophora* debris.

*A*. *adenophora* plants take too long in natural rotting because microbial activities related to the composting were greatly inhibited by allelopathic substances in *A*. *adenophora*. In our experiment, *Clostridium thermocellum* was employed to compost *A*. *adenophora* debris. Visual and microscopic examination proved that on-site composting *A*. *adenophora* plants enabled all the roots, stems, shoots, and seeds to be completely decayed. Besides, we need to make sure the degradation of all the plant-toxic allelochemicals in *A*. *adenophora* debris. To avoid detecting the toxicity of each plant-toxic allelochemicals one by one, a seed germination test can overally examine the phytotoxicity of *A*. *adenophora* debris. UCAE inhibited barley seed germination (except that low concentration UCAE increased the germination percentage) and decreased young seedling growth in contrast to CAE which behaved otherwise. These results confirmed the fact that *A*. *adenophora* contains allelochemicals harmful to plants, while the composed *A*. *adenophora* had already no negative allelopathic effect, only high-quality organic nutrients left for promoting plant growth.

During the seed imbibition process, embryos release gibberellin and then transfer this phytohormone to aleurone layers, inducing the synthesis of lytic enzymes such as proteinase, amylase, and phytase. As a result, protein, starch, and insoluble P (mainly inositol hexaphosphate) in endosperms are hydrolyzed into amino acids, glucose, and soluble phosphate for building up new plants [37]. The germinating seeds exposed to UCAE contained higher proteins, starches, and insoluble P, but lower free amino acids, sugars, and soluble P than those in the blank control treatment. In contrast, CAE exhibited an opposite trend to UCAE. These results suggested the inhibition of macromolecule hydrolysis by allelochemicals in *A*. *adenophora* and degradation of these harmful substances by on-site composting. Jiao et al. [18] found that CA was rich in humic acid, a phytohormone-like compound, an appropriate pH (7.12) suitable for plants and soils, and higher contents of nitrogen and potassium, which could explain why CAE significantly promoted barley seed germination and young seedling growth.

Dehydrogenase activity reflects energy metabolism in roots, influencing plant nutrient uptake [38]. Nitrate reductase catalyzes NO_3_^-^ to form NH_3_ and thus the activity of this enzyme is important for N assimilation [37]. Chlorophyll, a pigment to absorb solar energy in photosynthesis, greatly influences the photosynthesis rate [39]. Yu et al. [40] found that phytotoxicity of allelochemicals releasing from *A*. *adenophora* was characterized by reducing activity of dehydrogenation and nitrate reductase, as well as chlorophyll concentration in neighboring plants. Thus, the decrease in the three indexes by UCAE may lead to the poor growth and development of young seedlings.

Applying UCA negatively influenced soil enzyme activities, as well as soil microbial biomass carbon and nitrogen concentrations. Higher soil enzyme activities in CF+CA is probably due to the improvement of soil conditions, accelerating microbes in producing more enzymes and enzymatic reactions [41]. Meanwhile, higher soil enzyme activities promoted organic matter recycling, and nutrients mobilization [42]. Higher soil dehydrogenases activity of CF+CA means that there are abundant live microbes in the soils [43]. Most importantly, the variation of soil enzyme activities are closely related to soil health and quality [44]. Consequently, CF+CA enhanced the soil quality as indicated by the increased soil enzyme activities.

CF+CA was of higher quantity of 16S rDNA sequences, richness and diversity of bacterial communities in soil, which suggested that the secondary metabolites released by *A*. *adenophora* was lower enough to be no harm to soil microbes, which was in accordance with that concluded by Delgado-Baquerizo et al. [45].

Compared with CF, CF+CA significantly increased plant nutrients uptake (including N, P, and K) and yield, and improved grain quality in contrast to CF+UCA which behaved oppositely. These results confirmed once again that *A*. *adenophora* contained allelochemicals harmful to other plants and on-site composting decomposed these allelochemicals in practical crop cultivation. CA may thus have similar functions as other organic fertilizers, including at least the decrease in the use of chemical fertilizers but an increase in the crop yield without quality sacrifice. Application of CA in combination with chemical fertilizers may ensure high sustainable soil productivity, fertility, and quality in agriculture. The on-site composting and using *A*. *adenophora* as an organic fertilizer may be an effective way to integrate control and utilization of the plant residues as a fertilizer resource.

## Conclusion

*A*. *adenophora* extracts contains phytotoxic allelochemicals, inhibiting barley seed germination, the mechanism may be the inhibition of bio-macromolecules hydrolysis (including protein, starch, and phytin) in endosperms. On-site composting was effective in eliminating DTD and HHO contained in *A*. *adenophora* debris, CAE promoted seed germination and seedling growth, increased chlorophyll level in the leaves, and stimulated the activities of dehydrogenase and nitrate reductase in plants, CF+CA enhanced barley plant nutrients uptake, and achieved higher barley yield without quality sacrifice, and also functioned positively in increasing soil enzyme activities (urease, invertase, dehydrogenase), microbial biomass (SMBN and SMBC), biodiversity and predominant microbial composition, while CF+UCA performed conversely. The findings in this paper deepened our understanding of *A*. *adenophora* utilization, and implied that on-site composting *A*. *adenophora* may be an effective approach for converting *A*. *adenophora* into a plant-friendly organic fertilizer, being undoubtedly worth advocating.

## Supporting information

S1 File (RAR)
